# The Clinical Manifestation Variety and Management Choice of Meckel's Diverticulum with Complication: A Single Center Experience

**DOI:** 10.1155/2021/6640660

**Published:** 2021-02-08

**Authors:** Tingliang Fu, Xiaoliang Xu, Lei Geng, Yanli Huang, Guojian Ding, Hong Ji

**Affiliations:** ^1^Department of Pediatric Surgery, Binzhou Medical University Hospital, Binzhou, Shandong 256603, China; ^2^Department of Surgery, Jinan Maternity and Child Care Hospital Affiliated to Shandong First Medical University, 3rd Jian-Guo Xiaojing Road, Jinan, Shandong 250000, China; ^3^Department of Pathology, Binzhou Medical University Hospital, Binzhou, Shandong 256603, China

## Abstract

**Background:**

The study was to analyze the clinical manifestation variety and management choices of symptomatic Meckel's diverticulum in children.

**Methods:**

From July 2008 to October 2018, 28 cases of Meckel's diverticulum with a variety of complications were admitted to our hospital. The clinical data included age, gender, symptoms and signs, investigations, intraoperative and pathological findings, and outcome.

**Results:**

The ratio of males to females was 2.5 : 1. The diagnoses were made by ^99m^Tc-pertechnetate scan (in 5 cases) and by exploratory laparotomy (in 2 cases). The initial diagnosis in the other cases includes intussusception (in 4 cases), acute appendicitis (in 5 cases), intestinal obstruction (unknown origin), peritonitis, and even shock in 12 cases. Laparoscopic surgery was performed in 8 cases; 18 cases underwent open surgery. Excision of partial bowel segment with diverticulum and primary anastomosis was done in 22 cases and wedge resection of diverticulum in 4 cases. Two other cases received nonoperative therapy and went to other hospitals to receive surgical management. Ectopic gastric mucosa in the diverticulum was found in 9 cases, including 6 cases with lower gastrointestinal bleeding.

**Conclusion:**

The clinical characteristics of Meckel's diverticulum varied. Children with hematochezia, peritonitis, and intestinal obstruction without history of prior abdominal operation should be suspected with this disease until proven otherwise. Hematochezia is often associated with ectopic gastric mucosa in the diverticulum. Laparoscopic surgery should be one of the choices for the diagnosis and treatment of Meckel's diverticulum with complications.

## 1. Introduction

Meckel's diverticulum is a congenital malformation caused by incomplete degeneration of the yolk sac in the early embryo, and the incidence is about 1.4% to 2% of the population as a whole [1]. But only about 2% of the population with Meckel's diverticulum present symptoms [2]. It can complicate with lower gastrointestinal bleeding, different types of intestinal obstruction, diverticulitis or perforation, and carcinoid or adenocarcinoma [3]. Intestinal obstruction is a more common complication in adults, whereas in children, bleeding is the more common complication [4]. It is key to make early diagnosis and give prompt and appropriate treatment for children presenting acute abdomen associated with Meckel's diverticulum. Any delayed diagnosis and treatment may lead to serious consequences, even life threatening. The present observation was to analyze the clinical data and experience of Meckel's diverticulum in a single medical center and to emphasize its variety of clinical manifestations and rational management choices so as to improve the clinical outcome of patients with this unusual disorder.

## 2. Methods

From July 2008 to October 2018, 28 cases with Meckel's diverticulum were admitted to our hospital. The diagnosis was confirmed by ^99m^Tc-pertechnetate scan, ultrasound, surgical exploration, and histopathological examination. The clinical data included age, gender, symptoms, investigation, surgical procedure, and clinical outcome. The existence of ectopic gastric mucosa was evaluated in the resected diverticulum specimen.

## 3. Results

### 3.1. Clinical Manifestations

**Figure 1:**
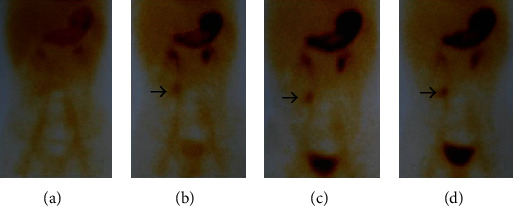
A 12-year-old boy with massive lower gastrointestinal bleeding; ^99m^Tc-pertechnetate scan was positive. The arrows show the existence of ectopic gastric mucosa.

The ratio of males to females was 2.5 : 1. The clinical manifestations of symptomatic Meckel's diverticulum included lower gastrointestinal bleeding, intussusception, intestinal volvulus, abdominal internal hernia, adhesive bowel obstruction, diverticulitis, perforation, shock, etc. There were 8 cases with lower gastrointestinal bleeding, and five were diagnosed by ^99m^Tc-pertechnetate scan (Figure 1).

There were 11 cases with intestinal obstruction, including intussusception in 4, entrapment or internal hernia in 6, and bowel necrosis due to volvulus in one case. Six cases with peritonitis due to diverticulitis or perforation were diagnosed by laparotomy.

### 3.2. Surgical Procedure and Clinical Outcome

Laparoscopic exploration was performed in 8 cases. Once the diagnosis was made, a semicircle incision around the umbilicus was done to exposure the diverticulum and to excise it with an adjacent bowel segment. Open surgery was performed in 18 cases, including cuneiform resection or bowel segment excision and primary anastomosis with good outcome.

### 3.3. Pathological Findings

**Figure 2:**
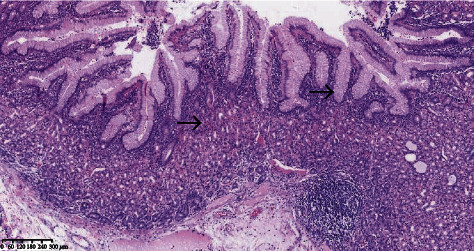
Ectopic gastric mucosa was found in Meckel's diverticulum; the arrows show the ectopic gastric mucosa.

In our case series, 9/19 (47.37%) cases with ectopic gastric mucosa in the diverticulum were detected by pathological examination, including 6/8 (75%) cases with intestinal bleeding (Figure 2). Two other cases with diverticulitis or gastrointestinal bleeding were diagnosed by ultrasound and transferred to other hospitals to undergo surgical management. Ectopic gastric mucosa could not be identified due to necrotic diverticulum or other reasons in 7 cases.

## 4. Discussion

### 4.1. Gender and Age Distribution

Lee et al. [5] reported that the ratio of males to females who suffered from Meckel's diverticulum associated with complications is about 2.3 : 1, while Rho et al. [6] reported that the ratio is 7.5 : 1. In the present study, the ratio was 2.5 : 1. Mackey and Dineen [7] reported that there is no gender difference in the population with asymptomatic Meckel's diverticulum. In general, the incidence of Meckel's diverticulum associated with complication is more common in males than in females [8]. The high peak of the occurrence is patients under 5 years of age [9]. Other studies reported that 66.5%-75% of the patients are under 10 years of age [6, 8, 10]. In our case series, 79% of symptomatic cases were younger than 10 years of age.

### 4.2. Pathological Features

The incidence of ectopic gastric tissue in symptomatic Meckel's diverticulum is 45%~80% [11]. The length of Meckel's diverticulum and the location of the ectopic gastric mucosa play an important role in surgical management [12, 13]. In symptomatic Meckel's diverticulum, ectopic gastric mucosa usually locates in the basal part, so diverticulectomy without an associated bowel segment may be insufficient. The ectopic gastric mucosa can be found in different parts of the diverticulum or scattered in small islands [14]. Ectopic pancreatic tissue is usually located at the top of the diverticulum and is yellowish-white, granular, so it is easy to identify [15]. In our case series, 9/19 (47.37%) cases with ectopic gastric mucosa were detected by pathological examination, especially in patients with lower gastrointestinal bleeding (75%).

### 4.3. The Many Faces of Clinical Representations

The variety of clinical manifestations in patients with symptomatic Meckel's diverticulum including small intestinal obstruction, intussusception, inflammation, lower gastrointestinal hemorrhage, perforation, and other rare conditions was reported [4, 9, 16, 17]. Ectopic gastric mucosa is present in 90% of cases of Meckel's diverticulum with hemorrhage. In our case series, ectopic gastric mucosa was detected in 75% of the patients with lower gastrointestinal bleeding, while those without gastrointestinal hemorrhage, only 16.7%. Diverticulum with an abnormal cord causes intestinal volvulus, internal hernia, intussusception, and other rare disorders, such as twisted intestine, presenting small bowel obstruction without history of prior abdominal surgery. It is usually difficult to make preoperative diagnosis of inflammation of Meckel's diverticulum [18]. From the present group of cases, lack of awareness and the development of nonspecific symptoms may make the diagnosis of Meckel's diverticulum difficult. Especially in patients with unexplanatory shock, an acute abdomen should be suspected until proven otherwise. In a word, early and correct clinical diagnosis is based on a high index of suspicion [19].

### 4.4. Surgical Treatment Choice

In the era of minimally invasive surgery, laparoscopic diverticulectomy for Meckel's diverticulum is one of the choices. But, in the case with short and wide basal diverticulum, laparoscopic resection and anastomosis could become difficult. Onen et al. [10] suggested that if the base of diverticulum is too large, or if there is an ulcer on the mesentery side, the diverticulum and adjacent ileum segment should be excised and primary end-to-end anastomosis should be performed. At present, laparoscopic exploration should be one of the preferred approaches for the diagnosis and management of Meckel's diverticulum [11, 20]. However, it is difficult to determine whether there is a mass or thick base in the diverticulum and to evaluate the presence of ectopic gastric mucosa, so it is easier for surgeons to choose laparoscopy-assisted surgery [21]. Gezer et al. [17] found that touching the tissue under laparoscopy can mislead the existence or absence of ectopic gastric mucosa. In the present case series, precise diagnosis before operation was not made in 8 cases with Meckel's diverticulum associated with complications. The diagnosis of Meckel's diverticulum was made by laparoscopic exploration. Meckel's diverticulum cuneiform resection or Meckel's diverticulum plus adjacent segment resection with primary intestinal anastomosis was undergone with good outcome.

In conclusion, patients with Meckel's diverticulum may present variable symptoms and pathological findings. In children, hematochezia or intestinal obstruction without history of prior abdominal operation should be considered in the differential diagnosis of Meckel's diverticulum. Meckel's diverticulum complicated with lower gastrointestinal bleeding is usually related to the existence of ectopic gastric mucosa. ^99m^Tc-pertechnetate scan should be selected for the differential diagnosis of Meckel's diverticulum with lower gastrointestinal bleeding. Laparoscopic exploration and management should be one of the choices.

## Data Availability

The data used to support the findings of the retrospective analysis could be available from the corresponding authors upon request.
